# Rapamycin sensitizes cancer cells to growth inhibition by the PARP inhibitor olaparib

**DOI:** 10.18632/oncotarget.19667

**Published:** 2017-07-28

**Authors:** Atsushi Osoegawa, Joell J. Gills, Shigeru Kawabata, Phillip A. Dennis

**Affiliations:** ^1^ Department of Oncology, Johns Hopkins University School of Medicine, Baltimore, MD 21205, USA

**Keywords:** BRCA mutation, synthetic lethality, DNA damage response, SRB assay, cell cycle

## Abstract

Poly (ADP-ribose) polymerase inhibitors (PARPi) have been developed and tested in a context of combining it with double-stranded (ds) DNA repair defects or inhibitors, as PARP inhibitor impairs single-stranded (ss) DNA break repair, resulting in the activation of the dsDNA break repair machinery. Rapamycin has been widely prescribed for more than a decade and recent studies have revealed that it may inhibit dsDNA break repair. The combination of the PARP inhibitor olaparib and rapamycin synergistically inhibited cell proliferation in non-small cell lung cancer (NSCLC) cells, and even in triple negative breast cancer (TNBC) cells with BRCA1 mutations. Rad51, which forms a polymer on ssDNA upon dsDNA breaks, plays an essential role in homologous recombination. Olaparib induced Rad51 focus formation, while rapamycin successfully inhibited it both *in vivo* and *in vitro*, suggesting that this combination worked through the blocking of both ssDNA break repair and dsDNA break repair; hence the cells cannot go through the G2/M checkpoint. The protein level of PARP was a predictive marker for both PAR activity and Rad51 focus formation in this combination. Collectively, these data suggest that this combination could have therapeutic potential in the treatment of cancer with high PARP expression, or in combination with cytotoxic chemotherapy or radiotherapy.

## INTRODUCTION

Rapamycin, an allosteric inhibitor of the mechanical target of rapamycin (mTOR), was approved as an immune-suppressant in 1999 and has been widely prescribed for more than a decade. In recent years, interest has focused on its potential as an anticancer drug [1, 2]. The mTOR pathway is upregulated in various cancers [3, 4]. Lung and breast cancers are two major cancers with frequent PI3K-Akt-mTOR pathway alterations [5–7]. In the United States, approximately 450,000 (27.6% of all cancer cases) new cases and 200,000 (34.6% of total cancer) deaths occur due to lung and breast cancer each year [8]. Thus, there is an urgent need to develop novel therapies for these diseases. Rapamycin has been shown to impair tumor growth in several animal models by inhibiting the mTOR pathway [9–11]. However, clinical trials using rapamycin as a single agent failed to show significant results [12–14]. A recent study revealed a novel role of two mTOR inhibitors, rapamycin [15] and everolimus, [16] in the inhibition of homologous recombination (HR) and that they acted as a radio-sensitizer. Furthermore, many studies have suggested that rapamycin and irradiation can have synergistic effects [17, 18]. Rapamycin has also been shown to overcome resistance to poly (ADP-ribose) polymerase (PARP) inhibitor in BRCA1-deficient cancers [19]. In that study, the resistance could possibly be caused by restored HR function. However, the mechanism how rapamycin is involved in the DNA damage response (DDR) remains unclear.

PARP inhibitors were developed based on the concept that they could lead cancer cells to apoptosis when the cancer cells lack HR enzymes, including BRCA1/2, PTEN, CtIP and ARID1A [20–26]. According to this theory, when used in combination with HR inhibitors, including ATM inhibitor [27] and CDK inhibitor, PARP inhibitor successfully led cancer cells to synthetic lethality [28, 29]. We therefore hypothesized that rapamycin would sensitize cancer cells to growth inhibition induced by the PARP inhibitor, olaparib.

## RESULTS

### Rapamycin and olaparib synergistically inhibit the proliferation of NSCLC and TNBC cells

**Figure 1:**
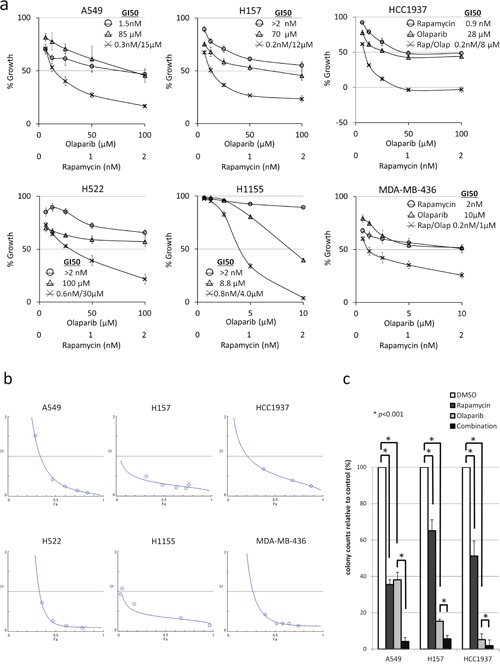
Rapamycin and olaparib synergistically inhibited the proliferation of NSCLS and TNBC cells **(a)** The growth inhibition by rapamycin (○), olaparib (Δ), or the combination of rapamycin and olaparib (×). NSCLC and TNBC cells were treated with drugs at the indicated concentrations for 48 h (A549, H157) or 72 h (H522, H1155, HCC1937 and MDA-MB-436). Bars, S.D. **(b)** The computer-simulated Fa-CI curves displayed synergism (CI<1), additive effects (CI=1), or antagonism (CI>1) for the entire spectrum of effect levels with the combination of rapamycin and olaparib. ○ The actual Fa-CI plot based on the experimental values. **(c)** A549, H157 or HCC1937 cells were treated with 0.5% DMSO, 1 nM rapamycin, olaparib (20 μM for A549 and 5 μM for H157 and HCC1937), or the combination every 3 days for 9 days. The colony counts are relative to DMSO-treated cells. Rap, rapamycin; Olap, olaparib; bars, S.D.

To assess the effects of drugs on cellular proliferation, rapamycin and olaparib were tested at various concentrations with a constant ratio of 1:50,000 for A549, H157, HCC1937, and H522, and 1:5,000 for H1155 and MDA-MB-436. With the exception to the 50% growth inhibition (GI50) of H1155, the GI50s obtained by the single administration of rapamycin were similar, throughout all of the tested cell lines, to those of previous reports [30]. On the other hand, the GI50s of single agent olaparib differed from cell line to cell line, with H1155 and MDA-MB-436 showing high sensitivity (GI50: 10 μM), H157 and HCC1937 showing moderate sensitivity (GI50: 35 μM and 70 μM, respectively), and A549 and H522 showing low sensitivity (GI50: 100 μM) (Figure 1a). The combination of rapamycin and olaparib decreased proliferation to a greater extent than either of the drugs alone in six tested cell lines (Figure 1a). To evaluate their synergistic effects, combination indices (CIs) were calculated using the CalcuSyn software program. The computer-simulated fraction affected (Fa)-CI curves showed synergism (CIs <1) in the six evaluated cell lines (Figure 1b). A clonogenic assay was performed to assess this drug combination in long-term treatment. The combination of rapamycin and olaparib inhibited clonogenicity to a greater extent than either of the drugs alone in three of the tested cell lines (Figure 1c). Note that when the two drugs were used in combination, lower concentrations of olaparib (20 μM for A549, 5 μM for H157 and HCC1937), which were clinically achievable, were required to achieve more than 95% inhibition. These data show that the combination of rapamycin and olaparib synergistically decreased cellular proliferation.

### PAR activity is increased by rapamycin and inhibited by olaparib

**Figure 2:**
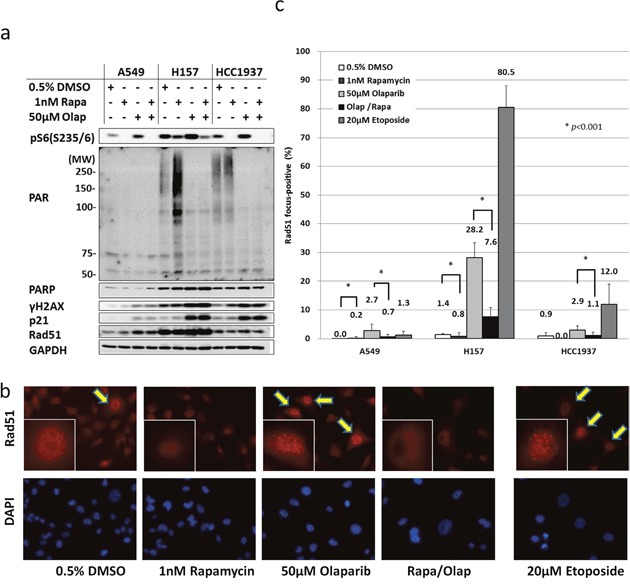
Rapamycin inhibits the induction of HR by olaparib **(a)** Olaparib inhibits the PAR activity enhanced by rapamycin. A549, H157 and HCC1937 cells were treated with either DMSO, 1 nM rapamycin, 50 μM olaparib, or the combination of rapamycin and olaparib for 4h. Immunoblotting was performed. **(b)** The inhibition of Rad51 foci by rapamycin. Cells were treated with the drugs at the concentration described in **(a)** for 48 h. Rad51 focus formation was assessed based on immunofluorescence. Arrows, Rad51 focus-positive cells. Representative nucleus images are shown in the boxes. **(c)** Rad51 focus-positive cells were counted. Columns, the mean count from at least five high-power fields. Bars, S.D. ^*^p<0.001.

To evaluate the underlying mechanism, poly (ADP-ribose) (PAR) activity and DDR enzymes were examined by Western blotting. The base level of PAR activity was low in A549 and high in H157 and HCC1937, which was positively correlated with the protein level of PARP. Interestingly, rapamycin increased the PAR activities in H157 and HCC1937, suggesting that the single-stranded DNA (ssDNA) damage repair mechanism was activated by the compensation of HR inhibition caused by rapamycin. However, the protein levels of Rad51, which is used as a marker for HR, did not change after rapamycin treatment. Olaparib completely inhibited the PAR activities, and caused the activation of the DDR enzyme, γH2AX (Figure 2a). DDR also increased the p21 level, leading to the accumulation of G2/M phase (Supplementary Figure 1).

### The inhibition of Rad51 focus formation by rapamycin was activated by the DDR

After it was revealed that Rad51 protein levels from whole cell lysates did not change in response to rapamycin treatment, we analyzed Rad51 focus formation by immunofluorescence microscopy (Figure 2b). Rad51 foci were formed by olaparib treatment. The rate of focus formation was higher in H157 cells and lower in A549 cells, which correlated with the magnitude of DDR induced by olaparib that had been determined by Western blotting (Figure 2a). Surprisingly, rapamycin significantly inhibited Rad51 focus formation (73% in H157, 74% in A549 and 62% in HCC1937), almost to the baseline level. Rapamycin therefore inhibited HR in the three tested cell lines (Figure 2c).

### The combination of olaparib and rapamycin inhibits NSCLC and TNBC tumor growth *in vivo*

**Figure 3:**
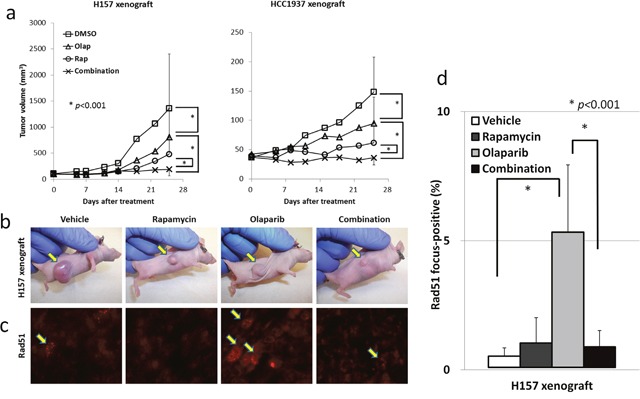
The combination of rapamycin and olaparib inhibits NSCLC and TNBC tumor growth *in vivo* **(a)** H157 cells (left) and HCC1937 cells (right) were grown as xenografts in athymic NCr-nu/nu. Bars, S.D. ^*^p<0.001. **(b)** Representative photos of H157 xenograft tumors after 25 days of either treatment. Arrows, H157 tumors. **(c)** Rad51 focus formation *in vivo*. H157 xenograft tumors after 5 days of treatment were excised and prepared for immunofluorescence microscopy as described in the Materials and Methods. **(d)** Rad51 focus-positive cells were counted. Columns, the mean from all four mice examined in H157 xenografts. Bars, S.D. ^*^p<0.001.

To determine whether the combination of rapamycin and olaparib enhances anti-tumor effects *in vivo*, athymic NCr-nu/nu mice bearing established H157 or HCC1937 tumor xenografts were treated with 1.5mg/kg rapamycin, 50mg/kg olaparib, or the combination of rapamycin and olaparib. The combination of rapamycin and olaparib was well tolerated without changes in the body weight of mice (Supplementary Figure 2), and resulted in an almost 85% decrease in H157 tumor growth by and an almost 75% decrease in HCC1937 tumor growth in comparison to vehicle-treated mice (Figure 3a, 3b). To investigate the correlation between the anti-tumor effects and the mechanisms identified *in vitro*, Rad51 focus formation was assessed in the tumors. Olaparib significantly increased Rad51 focus formation, and rapamycin significantly decreased Rad51 focus formation by nearly 85% (Figure 3c, 3d), suggesting that the combination of rapamycin and olaparib inhibited ssDNA damage repair and HR at the same time. Consequently, the tumor became incapable of repairing DNA damage *in vivo*.

## DISCUSSION

In the present study, we demonstrated for the first time that rapamycin sensitized NSCLC and TNBC cells to growth inhibition by olaparib through the inhibition of Rad51 focus formation. Emerging evidence from pre-clinical studies suggests that Rad51 is crucial for HR [31] and that its inhibition sensitizes tumors to PARP inhibitor. Cells that lack Rad51C, a Rad51 paralog, are reported to be highly sensitive to olaparib [32], and two murine breast cancer models have revealed that a pan-Class I PI3K inhibitor, BKM120, has the ability to inhibit Rad51, which was shown to lead to an increase in the anti-tumor effect of olaparib [33, 34]. Interestingly, the authors also showed the remnant Rad51 activities in BRCA-mutated tumors, indicating that PARP inhibition alone is not enough to expel BRCA-mutated cancers. Sun et al has reported that the resistance to olaparib in a BRCA1-mutated cell line was caused by restored HR function (as indicated by Rad51 foci formation), which could be overcome by rapamycin [19]. Our result expanded the possibility of olaparib and rapamycin combination further by showing that a low-dose of rapamycin could inhibit Rad51 focus-formation, not only in HR proficient NSCLC cells, but also in BRCA1-mutated TNBC cells by inhibiting the remnant HR mechanism. It is not surprising that the PI3K inhibitor BKM120 was able to inhibit ATM and its downstream substrates, which are essential for HR [35, 36], as ATM belongs to the PI3K-like kinase family. Unlike PI3K inhibitor, rapamycin is known to inhibit mTORC1, a downstream substrate of Akt [2]. Thus, rapamycin must inhibit Rad51 focus formation through a mechanism other than ATM inhibition. Sun et al has suggested that phosphorylation of S6 is responsible to the restored HR function, using an unphosphorylatable S6 knock-in cells [19]. Other study reported that the nuclear translocation of Rad51 is initiated by its phosphorylation at tyrosine 315 and 54 by c-Abl [37, 38]. mTOR is reported to inhibit starvation-specific gene expression by sequestering several nutrient-responsive transcription factors within the cytoplasm [39–41]. It is therefore possible that rapamycin inhibits Rad51 phosphorylation and its translocation into the nucleus. In fact, it has also been shown that RAD001 increases the nuclear expression of c-Abl [42], suggesting that mTOR inhibitor may inhibit Rad51 focus-formation through the nuclear retention of c-Abl. Further studies are warranted to clarify this mechanism.

Despite its promising mechanism, PARP inhibitors, including olaparib as a single drug, has failed to show clinical benefits in unselected cancer populations [43–45]. Thus far, olaparib was only successful in treating unselected targets in a maintenance trial for platinum-sensitive, relapsed, high-grade serous ovarian cancers in which the tumor burdens should be considerably small [46, 47]. Several clinical trials using drug combinations with PARP inhibitors are currently underway (NCT01623349, NCT01495351). The combination of PARP inhibitors with HR inhibitor (as used in the present study) has added a new venue for PARP inhibitor treatments. Considering the fact that approximately 40% of the patients who received BKM120 treatment experienced grade 3/4 toxicities [48] and that a low dose of rapamycin was used to accomplish the synergistic effect observed with this combination, the combination of rapamycin and olaparib may be more feasible than other drug combinations in the clinical setting. Thus, this combination could be tested in prevention, adjuvant, and maintenance therapies or with other modalities that cause further DNA damage, irradiation or cytotoxic chemotherapy.

The biomarker results represent another clinically significant finding of our study. It has been shown that Rad51 focus formation can be used as a marker of HR [49]. On the other hand, PARP expression and PAR activity have been used as markers of PARP inhibitor sensitivity [50–52]. Our study also demonstrated that both the PARP level and Rad51 focus formation could predict the treatment outcome of these drugs.

In summary, rapamycin and the PARP inhibitor olaparib synergistically inhibited NSCLC and TNBC cell growth. The underlying mechanism is as follows: olaparib increased HR through the inhibition of ssDNA damage repair and rapamycin inhibited HR at considerably low concentrations. This combination may expand the clinical indications for PARP inhibitors, and make therapeutic inroads in cancers that are difficult to treat, such as NSCLC and TNBC.

## MATERIALS AND METHODS

### Cell cultures

The NSCLC (A549, H157, H522 and H1155) cell lines were obtained as previously described [53]. TNBC (HCC1937 and MDA-MB-436) cells were gifts from Dr. Patricia S. Steeg (National Cancer Institute, Bethesda, MD, USA). These cells were maintained in RPMI1640 supplemented with 5% FBS. The genetic backgrounds of these cells are as follows; Kras mutations were present in A549 (G12S), H157 (G12R) and H1155 (Q61H); p53 mutations were present in H157 (E298^*^), H522 (P191fs^*^56), H1155 (R273H), HCC1937 (R306^*^) and MDA-MB-436 (ins 205); PTEN mutations were present in H157 (G251C), H1155 (R233^*^), HCC1937 and MDA-MB-436 (Homozygous deletion); and BRCA1 mutations were present in HCC1937 (Q1756fs^*^74) and MDA-MB-436 (5396+1G>A) [54].

### Reagents

Rapamycin and olaparib were obtained from LC Laboratories. The primary antibodies for the immunoblotting analyses for PARP, γH2AX, p21, p-S6 and GAPDH were purchased from Cell Signaling; PAR antibody was purchased from Enzo Life Sciences. Rad51 antibody was purchased from Santa Cruz Biotechnology.

### Cell proliferation assay

A549, H157 (10000 cells per well), H522, H1155, HCC1937 and MDA-MB-436 (20000 cells per well) cells were plated in 96-well plates and allowed to grow overnight. Rapamycin dissolved in DMSO, olaparib dissolved in DMSO, the combination rapamycin and olaparib dissolved in DMSO or DMSO alone was added, and the cells were allowed to grow for an additional 48 h or 72 h. Growth inhibition was determined by a sulforhodamine B assay [55, 56]. The percent growth value was calculated using the absorbance values of the untreated cells (on day 0 [D0]), DMSO-treated control cells (C), and drug-treated cells (T) as follows: (T−D0)/(C−D0) × 100 for concentrations in which T was ≥D0; or (T−D0)/C × 100 for concentrations in which T was <D0. The percent growth curve was generated on the basis of the % growth values in a dose-dependent manner. The experiments were performed in triplicate, and each drug concentration was evaluated in six wells for any given experiment. The CI is a quantitative measure of the degree of drug interaction, in terms of synergism (CI<1), additive effect (CI=1), or antagonism (CI>1), for a given endpoint of the effect measurement [57]. An additive effect is defined as the combined effect predicted by the mass-action law principle, synergism is defined as the production of a greater-than-expected additive effect, and antagonism is defined as the production of a smaller-than-expected additive effect. The CIs were calculated using the CalcuSyn software program (BIOSOFT, Cambridge, UK). The Fa is defined as a function (e.g., inhibition) of the effect level by a dose of a drug. Fa values were calculated according to the program's instructions as follows: (100−% growth value)/100, which indicated a growth inhibition value.

### Western blotting

Cells (5 × 10 [5] cells per well) were plated in six-well plates. The following day, the cells were treated with the drug or an equal volume of DMSO for the indicated times, and lysed in 2 × lysis buffer as described previously [53]. Cell lysates with equal amounts of protein were separated by SDS-PAGE, and then transferred to nitrocellulose membranes. The membranes were blocked for 1 h in blocking buffer (1 × TBS, 5% milk, 0.1% Tween 20) and placed in primary antibody diluted in 1 × TBS, 5% bovine serum albumin (BSA), 0.1% Tween 20, overnight at 4°C. The following day, the membranes were washed three times in wash buffer (0.1% Tween 20, 1 × TBS). The primary antibody was detected using horseradish-peroxidase-linked secondary antibodies, and visualized with an enhanced chemiluminescent detection system (Amersham Biosciences, Pittsburgh, PA, USA). The immunoblotting experiments were performed at least three times.

### Clonogenic assay

A long-term clonogenic assay was performed as previously described [21, 58]. A549, H157 (500 cells per well) and HCC1937 (2000 cells per well) cells were plated in six-well plates. The following day, treatment with the indicated drug(s) or DMSO was started and continued for nine days, with the medium and drugs replaced every three days. After 10-14 days, the cells were fixed and stained with sulforhodamine B. Colonies were counted using the ImageJ software program (National Institutes of Health).

### Immunofluorescent staining

The cells were plated in 96-well plates and allowed to grow overnight. The following day, the cells were treated with the drug(s) or an equal volume of DMSO for the indicated times. The medium was then removed, and the plates were rinsed once in PBS and fixed in 4% paraformaldehyde in PBS for 20 min. The cells were rinsed with PBS and permeabilized by treatment with PBS containing 0.3% Triton X-100 (PBS-T) for 10 min. After blocking with 1% BSA in PBS-T for 30 min, the cells were incubated with primary antibody overnight at 4°C. The cells were rinsed three times with PBS-T, incubated for 1 h at room temperature with Alexa 568 goat anti- Rabbit IgG antibody (Molecular Probes) at a concentration of 1:1000 and then rinsed three times in PBS-T. Antibodies were diluted in PBST containing 1% bovine serum albumin. DNA was stained with 100ng/ml DAPI (Molecular Probes). Images were obtained using an AMG EVOS fl Digital inverted fluorescence microscope. The frequencies of cells containing Rad51 foci were determined in at least five separate randomly selected high-power fields. At least 100 nuclei were counted per field. Nuclei containing more than 10 foci were classified as positive. For H157 xenografts, tumors were embedded in OCT, snap frozen and 4-μm sections were cut, stained and analyzed as described above.

### Drug treatment *in vivo*

Six-week-old female athymic NCr-nu/nu mice (Charles River Labs, Frederick, MD, USA) were subcutaneously injected in both rear flanks with 5 × 10 [6] H157 or HCC1937 cells in 50 *μ*l PBS and 50 *μ*l BD Matrigel Basement Membrane Matrix (BD Biosciences). When the transplanted tumors reached a volume of 50 mm [3], the mice were divided into the following four groups: intraperitoneal injection of either (1) vehicle (4% DMSO, 5% PEG, 5% Tween 80 in saline; once daily on days 1–5, 8–12, 15–19 and 22-25); (2) 50 mg/kg olaparib (once daily on days 1–5, 8–12, 15–17 and 22-25); (3) 1.5 mg/kg rapamycin (every other day) after a loading dose of 4.5mg/kg on the first day [59]; and (4) the combination of olaparib and rapamycin (the same dosing schedule that was used for each of the drugs). Animal weights and tumor measurements were made twice a week. Tumor volume was calculated using the following formula: *v*=(*ab*^2^)/2, where *a* is the long axis and *b* is the short axis. To examine the effect of the combination of olaparib and rapamycin on biomarkers, athymic NCr-nu/nu mice bearing H157 tumor xenografts were administered vehicle, olaparib, rapamycin, or the combination of olaparib and rapamycin at the above-described doses. After 5 days of treatment, the mice were sacrificed and the tumors were harvested for an analysis. All of the mice were housed in accordance with the guidelines of the Animal Care and Use Committee under an approved animal protocol.

### Statistics

The data are presented as the mean ± SD. Statistical significance between groups was determined using the Mann-Whitney U test. P values of less than 0.05 were considered to indicate statistical significance.

## SUPPLEMENTARY FIGURES



## Abbreviations

mTOR: mechanical target of rapamycin
HR: homologous recombination
PARP: poly (ADP-ribose) polymerase
DDR: DNA damage response
GI50: growth inhibition of 50%
CI: combination index
Fa: fraction affected
PAR: poly (ADP-ribose)
ssDNA: single-stranded DNA
dsDNA: double-stranded DNA
NSCLC: non-small cell lung cancer
TNBC: triple-negative breast cancer
BSA: bovine serum albumin

## References

[1] Guertin DA, Sabatini DM Defining the role of mTOR in cancer. Cancer Cell.

[2] Sabatini DM mTOR and cancer: insights into a complex relationship. Nat Rev Cancer.

[3] Guertin DA, Sabatini DM An expanding role for mTOR in cancer. Trends Mol Med.

[4] Hollander MC, Blumenthal GM, Dennis PA PTEN loss in the continuum of common cancers, rare syndromes and mouse models. Nat Rev Cancer.

[5] Tsurutani J, Fukuoka J, Tsurutani H, Shih JH, Hewitt SM, Travis WD, Jen J, Dennis PA Evaluation of two phosphorylation sites improves the prognostic significance of Akt activation in non-small-cell lung cancer tumors. J Clin Oncol.

[6] Tsao AS, McDonnell T, Lam S, Putnam JB, Bekele N, Hong WK, Kurie JM Increased phospho-AKT (Ser(473)) expression in bronchial dysplasia: implications for lung cancer prevention studies. Cancer Epidemiol Biomarkers Prev.

[7] Engelman JA Targeting PI3K signalling in cancer: opportunities, challenges and limitations. Nat Rev Cancer.

[8] Siegel R, Naishadham D, Jemal A Cancer statistics, 2012. CA Cancer J Clin.

[9] Geoerger B, Kerr K, Tang CB, Fung KM, Powell B, Sutton LN, Phillips PC, Janss AJ Antitumor activity of the rapamycin analog CCI-779 in human primitive neuroectodermal tumor/medulloblastoma models as single agent and in combination chemotherapy. Cancer Res.

[10] Ito D, Fujimoto K, Mori T, Kami K, Koizumi M, Toyoda E, Kawaguchi Y, Doi R In vivo antitumor effect of the mTOR inhibitor CCI-779 and gemcitabine in xenograft models of human pancreatic cancer. Int J Cancer.

[11] Amornphimoltham P, Patel V, Sodhi A, Nikitakis NG, Sauk JJ, Sausville EA, Molinolo AA, Gutkind JS Mammalian target of rapamycin, a molecular target in squamous cell carcinomas of the head and neck. Cancer Res.

[12] Galanis E, Buckner JC, Maurer MJ, Kreisberg JI, Ballman K, Boni J, Peralba JM, Jenkins RB, Dakhil SR, Morton RF, Jaeckle KA, Scheithauer BW, Dancey J Phase II trial of temsirolimus (CCI-779) in recurrent glioblastoma multiforme: a North Central Cancer Treatment Group Study. J Clin Oncol.

[13] Chang SM, Wen P, Cloughesy T, Greenberg H, Schiff D, Conrad C, Fink K, Robins HI, De Angelis L, Raizer J, Hess K, Aldape K, Lamborn KR Phase II study of CCI-779 in patients with recurrent glioblastoma multiforme. Invest New Drugs.

[14] Atkins MB, Hidalgo M, Stadler WM, Logan TF, Dutcher JP, Hudes GR, Park Y, Liou SH, Marshall B, Boni JP, Dukart G, Sherman ML Randomized phase II study of multiple dose levels of CCI-779, a novel mammalian target of rapamycin kinase inhibitor, in patients with advanced refractory renal cell carcinoma. J Clin Oncol.

[15] Chen H, Ma Z, Vanderwaal RP, Feng Z, Gonzalez-Suarez I, Wang S, Zhang J, Roti Roti JL, Gonzalo S, Zhang J The mTOR inhibitor rapamycin suppresses DNA double-strand break repair. Radiat Res.

[16] Mo W, Liu Q, Lin CC, Dai H, Peng Y, Liang Y, Peng G, Meric-Bernstam F, Mills GB, Li K, Lin SY mTOR inhibitors suppress homologous recombination repair and synergize with PARP inhibitors via regulating SUV39H1 in BRCA-proficient triple-negative breast cancer. Clin Cancer Res.

[17] Ekshyyan O, Rong Y, Rong X, Pattani KM, Abreo F, Caldito G, Chang JK, Ampil F, Glass J, Nathan CO Comparison of radiosensitizing effects of the mammalian target of rapamycin inhibitor CCI-779 to cisplatin in experimental models of head and neck squamous cell carcinoma. Mol Cancer Ther.

[18] Eshleman JS, Carlson BL, Mladek AC, Kastner BD, Shide KL, Sarkaria JN Inhibition of the mammalian target of rapamycin sensitizes U87 xenografts to fractionated radiation therapy. Cancer Res.

[19] Sun CK, Zhang F, Xiang T, Chen Q, Pandita TK, Huang Y, Hu MC, Yang Q Phosphorylation of ribosomal protein S6 confers PARP inhibitor resistance in BRCA1-deficient cancers. Oncotarget.

[20] Forster MD, Dedes KJ, Sandhu S, Frentzas S, Kristeleit R, Ashworth A, Poole CJ, Weigelt B, Kaye SB, Molife LR Treatment with olaparib in a patient with PTEN-deficient endometrioid endometrial cancer. Nat Rev Clin Oncol.

[21] Mendes-Pereira AM, Martin SA, Brough R, McCarthy A, Taylor JR, Kim JS, Waldman T, Lord CJ, Ashworth A Synthetic lethal targeting of PTEN mutant cells with PARP inhibitors. EMBO Mol Med.

[22] Turner N, Tutt A, Ashworth A Hallmarks of ‘BRCAness’ in sporadic cancers. Nat Rev Cancer.

[23] Leung M, Rosen D, Fields S, Cesano A, Budman DR Poly(ADP-ribose) polymerase-1 inhibition: preclinical and clinical development of synthetic lethality. Mol Med.

[24] Fong PC, Boss DS, Yap TA, Tutt A, Wu P, Mergui-Roelvink M, Mortimer P, Swaisland H, Lau A, O'Connor MJ, Ashworth A, Carmichael J, Kaye SB Inhibition of poly(ADP-ribose) polymerase in tumors from BRCA mutation carriers. N Engl J Med.

[25] Wang J, Ding Q, Fujimori H, Motegi A, Miki Y, Masutani M Loss of CtIP disturbs homologous recombination repair and sensitizes breast cancer cells to PARP inhibitors. Oncotarget.

[26] Shen J, Peng Y, Wei L, Zhang W, Yang L, Lan L, Kapoor P, Ju Z, Mo Q, Shih IM, Uray IP, Wu X, Brown PH ARID1A deficiency impairs the DNA damage checkpoint and sensitizes cells to PARP inhibitors. Cancer Discov.

[27] Williamson CT, Muzik H, Turhan AG, Zamò A, O'Connor MJ, Bebb DG, Lees-Miller SP ATM deficiency sensitizes mantle cell lymphoma cells to poly(ADP-ribose) polymerase-1 inhibitors. Mol Cancer Ther.

[28] Johnson N, Li YC, Walton ZE, Cheng KA, Li D, Rodig SJ, Moreau LA, Unitt C, Bronson RT, Thomas HD, Newell DR, D'Andrea AD, Curtin NJ Compromised CDK1 activity sensitizes BRCA-proficient cancers to PARP inhibition. Nat Med.

[29] Alagpulinsa DA, Ayyadevara S, Yaccoby S, Shmookler Reis RJ A cyclin-dependent kinase inhibitor, dinaciclib, impairs homologous recombination and sensitizes multiple myeloma cells to PARP inhibition. Mol Cancer Ther.

[30] Granville CA, Memmott RM, Balogh A, Mariotti J, Kawabata S, Han W, Lopiccolo J, Foley J, Liewehr DJ, Steinberg SM, Fowler DH, Hollander MC, Dennis PA A central role for Foxp3+ regulatory T cells in K-Ras-driven lung tumorigenesis. PLoS One.

[31] Sonoda E, Sasaki MS, Buerstedde JM, Bezzubova O, Shinohara A, Ogawa H, Takata M, Yamaguchi-Iwai Y, Takeda S Rad51-deficient vertebrate cells accumulate chromosomal breaks prior to cell death. EMBO J.

[32] Min A, Im SA, Yoon YK, Song SH, Nam HJ, Hur HS, Kim HP, Lee KH, Han SW, Oh DY, Kim TY, O'Connor MJ, Kim WH, Bang YJ RAD51C-deficient cancer cells are highly sensitive to the PARP inhibitor olaparib. Mol Cancer Ther.

[33] Juvekar A, Burga LN, Hu H, Lunsford EP, Ibrahim YH, Balmañà J, Rajendran A, Papa A, Spencer K, Lyssiotis CA, Nardella C, Pandolfi PP, Baselga J Combining a PI3K inhibitor with a PARP inhibitor provides an effective therapy for BRCA1-related breast cancer. Cancer Discov.

[34] Ibrahim YH, Garcia-Garcia C, Serra V, Lunsford EP, Ibrahim YH, Balmañà J, Rajendran A, Papa A, Spencer K, Lyssiotis CA, Nardella C, Pandolfi PP, Baselga J PI3K inhibition impairs BRCA1/2 expression and sensitizes BRCA-proficient triple-negative breast cancer to PARP inhibition. Cancer Discov.

[35] Liu Q, Xu C, Kirubakaran S, Zhang X, Hur W, Liu Y, Kwiatkowski NP, Wang J, Westover KD, Gao P, Ercan D, Niepel M, Thoreen CC Characterization of Torin2, an ATP-competitive inhibitor of mTOR, ATM, and ATR. Cancer Res.

[36] Mukherjee B, Tomimatsu N, Amancherla K, Camacho CV, Pichamoorthy N, Burma S The dual PI3K/mTOR inhibitor NVP-BEZ235 is a potent inhibitor of ATM- and DNA-PKCs-mediated DNA damage responses. Neoplasia.

[37] Popova M, Shimizu H, Yamamoto K, Camacho CV, Pichamoorthy N, Burma S Detection of c-Abl kinase-promoted phosphorylation of Rad51 by specific antibodies reveals that Y54 phosphorylation is dependent on that of Y315. FEBS Lett.

[38] Slupianek A, Schmutte C, Tombline G, Nieborowska-Skorska M, Hoser G, Nowicki MO, Pierce AJ, Fishel R, Skorski T BCR/ABL regulates mammalian RecA homologs, resulting in drug resistance. Mol Cell.

[39] Beck T, Hall MN The TOR signalling pathway controls nuclear localization of nutrient-regulated transcription factors. Nature.

[40] Cardenas ME, Cutler NS, Lorenz MC, Di Como CJ, Heitman J The TOR signaling cascade regulates gene expression in response to nutrients. Genes Dev.

[41] Hardwick JS, Kuruvilla FG, Tong JK, Shamji AF, Schreiber SL Rapamycin-modulated transcription defines the subset of nutrient-sensitive signaling pathways directly controlled by the Tor proteins. Proc Natl Acad Sci U S A.

[42] Mancini M, Corradi V, Petta S, Martinelli G, Barbieri E, Santucci MA mTOR inhibitor RAD001 (Everolimus) enhances the effects of imatinib in chronic myeloid leukemia by raising the nuclear expression of c-ABL protein. Leuk Res.

[43] Gelmon KA, Tischkowitz M, Mackay H, Swenerton K, Robidoux A, Tonkin K, Hirte H, Huntsman D, Clemons M, Gilks B, Yerushalmi R, Macpherson E, Carmichael J, Oza A Olaparib in patients with recurrent high-grade serous or poorly differentiated ovarian carcinoma or triple-negative breast cancer: a phase 2, multicentre, open-label, non-randomised study. Lancet Oncol.

[44] Tutt A, Robson M, Garber JE, Domchek SM, Audeh MW, Weitzel JN, Friedlander M, Arun B, Loman N, Schmutzler RK, Wardley A, Mitchell G, Earl H Oral poly(ADP-ribose) polymerase inhibitor olaparib in patients with BRCA1 or BRCA2 mutations and advanced breast cancer: a proof-of-concept trial. Lancet.

[45] Audeh MW, Carmichael J, Penson RT, Friedlander M, Powell B, Bell-McGuinn KM, Scott C, Weitzel JN, Oaknin A, Loman N, Lu K, Schmutzler RK, Matulonis U Oral poly(ADP-ribose) polymerase inhibitor olaparib in patients with BRCA1 or BRCA2 mutations and recurrent ovarian cancer: a proof-of-concept trial. Lancet.

[46] Ledermann J, Harter P, Gourley C, Friedlander M, Vergote I, Rustin G, Scott C, Meier W, Shapira-Frommer R, Safra T, Matei D, Macpherson E, Watkins C Olaparib maintenance therapy in platinum-sensitive relapsed ovarian cancer. N Engl J Med.

[47] Ledermann JA, Harter P, Gourley C, Friedlander M, Vergote I, Rustin G, Scott C, Meier W, Shapira-Frommer R, Safra T, Matei D, Fielding A, Spencer S Overall survival in patients with platinum-sensitive recurrent serous ovarian cancer receiving olaparib maintenance monotherapy: an updated analysis from a randomised, placebo-controlled, double-blind, phase 2 trial. Lancet Oncol.

[48] Bendell JC, Rodon J, Burris HA, de Jonge M, Verweij J, Birle D, Demanse D, De Buck SS, Ru QC, Peters M, Goldbrunner M, Baselga J Phase I, dose-escalation study of BKM120, an oral pan-Class I PI3K inhibitor, in patients with advanced solid tumors. J Clin Oncol.

[49] Birkelbach M, Ferraiolo N, Gheorghiu L, Pfäffle HN, Daly B, Ebright MI, Spencer C, O'Hara C, Whetstine JR, Benes CH, Sequist LV, Zou L, Dahm-Daphi J Detection of impaired homologous recombination repair in NSCLC cells and tissues. J Thorac Oncol.

[50] Byers LA, Wang J, Nilsson MB, Fujimoto J, Saintigny P, Yordy J, Giri U, Peyton M, Fan YH, Diao L, Masrorpour F, Shen L, Liu W Proteomic profiling identifies dysregulated pathways in small cell lung cancer and novel therapeutic targets including PARP1. Cancer Discov.

[51] Rajan A, Carter CA, Kelly RJ, Gutierrez M, Kummar S, Szabo E, Yancey MA, Ji J, Mannargudi B, Woo S, Spencer S, Figg WD, Giaccone G A phase I combination study of olaparib with cisplatin and gemcitabine in adults with solid tumors. Clin Cancer Res.

[52] Herriott A, Tudhope SJ, Junge G, Rodrigues N, Patterson MJ, Woodhouse L, Lunec J, Hunter JE, Mulligan EA, Cole M, Allinson LM, Wallis JP, Marshall S PARP1 expression, activity and ex vivo sensitivity to the PARP inhibitor, talazoparib (BMN 673), in chronic lymphocytic leukaemia. Oncotarget.

[53] Gills JJ, Lopiccolo J, Tsurutani J, Shoemaker RH, Best CJ, Abu-Asab MS, Borojerdi J, Warfel NA, Gardner ER, Danish M, Hollander MC, Kawabata S, Tsokos M Nelfinavir, A lead HIV protease inhibitor, is a broad-spectrum, anticancer agent that induces endoplasmic reticulum stress, autophagy, and apoptosis in vitro and in vivo. Clin Cancer Res.

[54] Forbes SA, Bindal N, Bamford S, Cole C, Kok CY, Beare D, Jia M, Shepherd R, Leung K, Menzies A, Teague JW, Campbell PJ, Stratton MR, Futreal PA COSMIC: mining complete cancer genomes in the Catalogue of Somatic Mutations in Cancer. Nucleic Acids Res.

[55] Skehan P, Storeng R, Scudiero D, Monks A, McMahon J, Vistica D, Warren JT, Bokesch H, Kenney S, Boyd MR New colorimetric cytotoxicity assay for anticancer-drug screening. J Natl Cancer Inst.

[56] Kawabata S, Gills JJ, Mercado-Matos JR, Lopiccolo J, Wilson W, Hollander MC, Dennis PA Synergistic effects of nelfinavir and bortezomib on proteotoxic death of NSCLC and multiple myeloma cells. Cell Death Dis.

[57] Chou TC Theoretical basis, experimental design, and computerized simulation of synergism and antagonism in drug combination studies. Pharmacol Rev.

[58] Farmer H, McCabe N, Lord CJ, Tutt AN, Johnson DA, Richardson TB, Santarosa M, Dillon KJ, Hickson I, Knights C, Martin NM, Jackson SP, Smith GC, Ashworth A Targeting the DNA repair defect in BRCA mutant cells as a therapeutic strategy. Nature.

[59] Granville CA, Warfel N, Tsurutani J, Hollander MC, Robertson M, Fox SD, Veenstra TD, Issaq HJ, Linnoila RI, Dennis PA Identification of a highly effective rapamycin schedule that markedly reduces the size, multiplicity, and phenotypic progression of tobacco carcinogen-induced murine lung tumors. Clin Cancer Res.

