# Supplementary material for: Rapamycin sensitizes cancer cells to growth inhibition by the PARP inhibitor olaparib
Source: Oncotarget. 2017 Jul 28;8(50):87044–53. doi: 10.18632/oncotarget.19667 (PMC5675614; doi:10.18632/oncotarget.19667)
Supplement: Supplementary file 1 [file oncotarget-08-87044-s001.pdf]

Rapamycin sensitizes cancer cells to growth inhibition by the PARP inhibitor olaparib

SUPPLEMENTARY MATERIALS

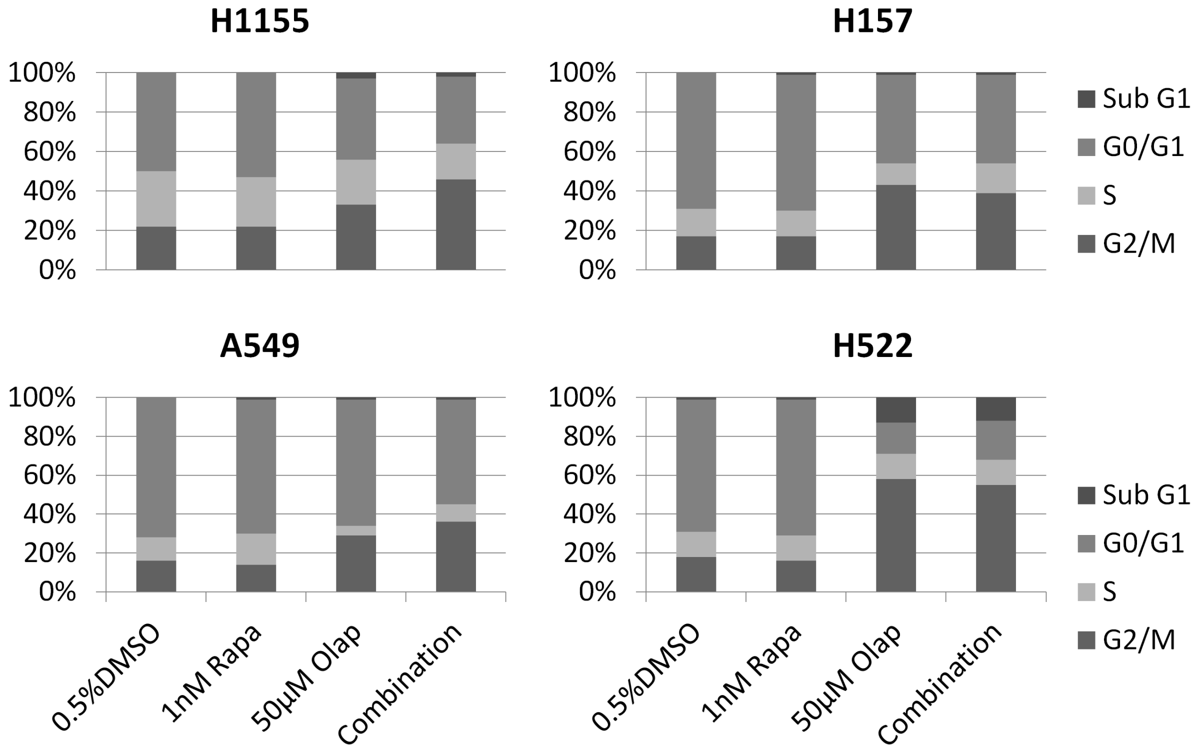

Supplementary Figure 1: Olaparib induced G2/M accumulation. Cells were plated in 6 well plates and allowed to grow overnight. The following day, cells were treated with indicated drugs or equal volume of DMSO for 48h (A549 and H157) or 72h (H1155 and H522), and then washed with ice cold PBS. Cells were then trypsinized and transferred into a polystylene tube for centrifuge. Cell pellets were then suspended and fixed in ice cold 70% methanol, stained with propidium iodide and subjected for flow cytometry using FACS LSRII (BD Biosciences) and analyzed using WinMDI Software (The Scripps Institute, Flow Cytometry Core Facility).

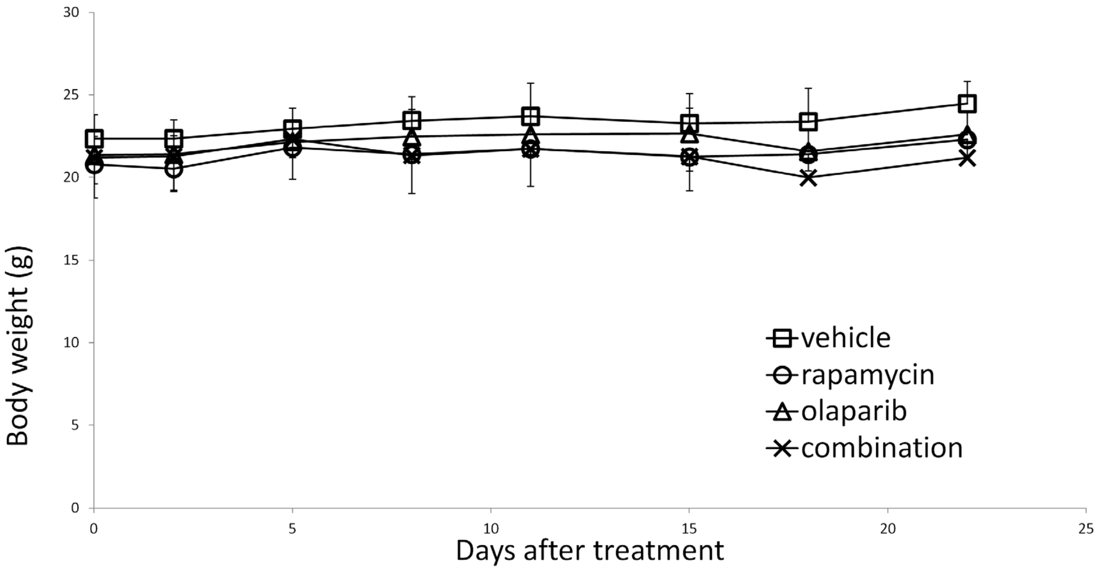

Supplementary Figure 2: Body weight changes in mice bearing tumor xenografts. HCC1937 cells were grown as xenografts in athymic NCr-nu/nu. Body weight changes were plotted in mice treated with indicated drugs as described in the Materials and Methods. Bars, S.D.
